# The effect of household heads training about the use of treated bed nets on the burden of malaria and anaemia in under-five children: a cluster randomized trial in Ethiopia

**DOI:** 10.1186/1475-2875-11-8

**Published:** 2012-01-06

**Authors:** Amare Deribew, Zewdie Birhanu, Lelisa Sena, Tariku Dejene, Ayalu A Reda, Morankar Sudhakar, Fessehaye Alemseged, Fasil Tessema, Ahmed Zeynudin, Sibhatu Biadgilign, Kebede Deribe

**Affiliations:** 1 Department of Epidemiology, Jimma University, Jimma, Ethiopia; 2 Department of Health Education and Behavioral Sciences, Jimma University, Jimma, Ethiopia; 3 Department of Public Health, College of Health Sciences, Haramaya University, Harar, Ethiopia; 4 Department of Medical Laboratory and Pathology, Jimma University, Jimma, Ethiopia; 5 Faculty of public Health, Jimma University, Jimma, Ethiopia

## Abstract

**Background:**

Long-lasting insecticide-treated bed nets (LLITN) have demonstrated a significant effect in reducing malaria-related morbidity and mortality. However, barriers on the utilization of LLITN have hampered the desired outcomes. The aim of this study was to assess the effect of community empowerment on the burden of malaria and anaemia in under-five children in Ethiopia.

**Methods:**

A cluster randomized trial was done in 22 (11 intervention and 11 control) villages in south-west Ethiopia. The intervention consisted of tailored training of household heads about the proper use of LLITN and community network system. The burden of malaria and anaemia in under-five children was determined through mass blood investigation at baseline, six and 12 months of the project period. Cases of malaria and anaemia were treated based on the national protocol. The burden of malaria and anaemia between the intervention and control villages was compared using the complex logistic regression model by taking into account the clustering effect. Eight Focus group discussions were conducted to complement the quantitative findings.

**Results:**

A total of 2,105 household heads received the intervention and the prevalence of malaria and anaemia was assessed among 2410, 2037 and 2612 under-five children at baseline, six and 12 months of the project period respectively. During the high transmission/epidemic season, children in the intervention arm were less likely to have malaria as compared to children in the control arm (OR = 0.42; 95%CI: 0.32, 0.57). Symptomatic malaria also steadily declined in the intervention villages compared to the control villages in the follow up periods. Children in the intervention arm were less likely to be anaemic compared to those in the control arm both at the high (OR = 0.84; 95%CI: 0.71, 0.99)) and low (OR = 0.73; 95%CI: 0.60, 0.89) transmission seasons.

**Conclusion:**

Training of household heads on the utilization of LLITN significantly reduces the burden of malaria in under-five children. The Ministry of Health of Ethiopia in collaboration with other partners should design similar strategies in high-risk areas to control malaria in Ethiopia.

**Trial registration:**

Australia and New Zealand Clinical Trials Register (ANZCTR): ACTRN12610000035022

## Background

Long-lasting insecticide-treated bed nets (LLITN) are proven to reduce malaria-related morbidity and mortality in under-five children [1-5]. However, barriers on the utilization of LLITN have hampered the desired outcomes. Much attention has been given to coverage and ensuring LLITN ownership although the actual problem goes beyond the mere availability and ownership of LLITN. In Ethiopia, the government distributed 20 million LLITN between 2005 and 2007, free-of-charge, to households with vulnerable groups [6,7]. Nonetheless, the actual utilization of LLITN in Ethiopia remains very low. A study conducted in the two biggest regions of Ethiopia showed that 91% households owned at least one LLITN and only 65% of the owned LLITN had been used the prior night [8]. A national survey report showed that there is considerable disparity between LLITN possession and use [9]. LLITN possession was shown to range between 0.1% and 28.5%, while utilization among under-five children ranged between 0% and 16%. The rate of utilization of LLITN declines at the beginning of the dry season [10].

Low community awareness and poor utilization of the preventive methods pose serious challenges for the malaria control programmes [11-13]. In Ethiopia, it was observed that many mothers had used LLITN for scarves and bed sheets to prevent lice and fleas and other purposes [9,14]. Studies conducted in Africa have attributed the low usage of LLITN to practical and technical difficulties related to the fixing of the net above the mat and the design of the house [9,15]. To translate the benefit of LLITN in the real situations, interventions that address behavioral aspect of LLITN utilization are crucial. The community should have access to information and skill-based trainings to properly use LLITN. Previous studies have not addressed behavioral aspect of LLITN utilization and its impact on malaria burden. Cognizant of this fact, a cluster randomized trial was conducted in southwest Ethiopia to assess the effect of training of the household heads on proper use of LLITN on the burden of malaria and anaemia in under-five children.

## Methods

### Study area

The study was conducted in south-west Ethiopia in Gilgel Gibe Field Research Centre (GGFRC), which is a malaria endemic area. The center was established in 2005 and is located 260 KM southwest of Addis Ababa, the capital city of Ethiopia. The GGFRC consisted of ten *kebeles *(smallest administrative units in Ethiopia) with a population of 50,000. Detail description of the study area is published elsewhere [16,17]. The prevalence of malaria in under-five children in GGFRC was high (8.3-10.5%) due to the possible ecological changes as a result of an artificial dam [16,17].

### Study design and sampling

A cluster randomized trial was done from February 2009 to February, 2010 in Gilgel Gibe Field Research Center, Southwest Ethiopia. The study population consisted of heads of the households and under-five children. From a total of 52 villages, 11 intervention and 11 control villages (*Gots*) were included in the study. The detail sampling technique and the trial profile is published elsewhere [16]. In brief, the sample size was determined based on the following assumptions: a prevalence of LLITN utilization (the primary outcome variable) of 27%, a 40% difference in the utilization of LLITN between the intervention and control villages, 95% CI, and 80% power. Eleven *Gots *in the north direction of the reservoir of Gilgel Gibe Dam were selected randomly as intervention whereas other 11 *Gots *in the south direction of the dam were selected to be control groups and both groups are within the same distance (10-15 Km) from the reservoir of the dam. All households in each cluster/*Gots *were included in the study. During the baseline survey, a total of 4135 households (2105 in the intervention village and 2030 in control villages) with a total population of 21,673 were included. Of the total population in the baseline survey, 2410 were under-five children. At six and 12 months of the project period, mass blood investigations in all under-five children were done in the same villages.

### The intervention

The intervention consisted of tailored training of heads of the households on the proper use of LLITN and establishing community network system. A total 2105 household heads in the intervention villages were trained how to use LLITN properly in a rural households. The training was given by nine village residents who received training of trainers (TOT) about utilization of LLITN. The training was supported by demonstration on the use of LLITN in real situation in the rural *Tukul *houses [16]. After the training, each household received at least two LLITN. The trained village residents then strictly followed the proper utilization of LLITN by under-five children in each household monthly using observation checklist [16]. During a household visit, they checked the status of the net, whether it was properly hanged and could reach under the bed/mattress in all directions.

**Figure 1:**
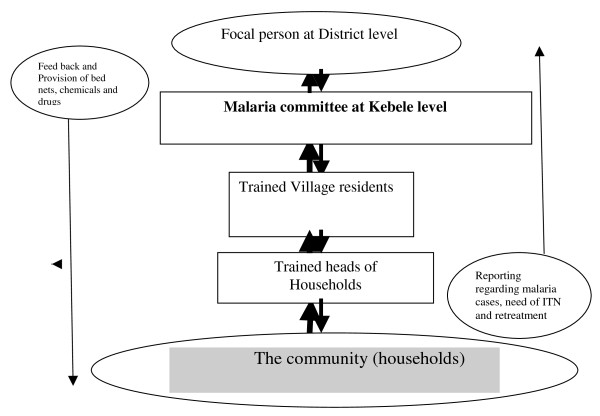
**The community network system for the intervention villages, Southwest Ethiopia**.

In the intervention villages, community network system was also established to monitor the utilization of LLITN and make the intervention sustainable (Figure 1). The network consisted of monthly meeting and discussion between key partners such as focal malaria expert at the district health office, malaria committee at the district health office, the trained village residents, and the researchers. The network ensured whether proper follow up of households were made and addressed major challenges such as shortage of LLITN and anti-malaria drugs. Like the intervention villages, each household in the control villages received at least two LLITN. However, the training and community network system were absent in the control villages.

### Measurement of burden of malaria and anaemia

To monitor the burden of malaria in under-five children, mass blood investigation in the intervention and control villages were done at baseline, six and 12 months of the project period. The mass blood investigation was made at the center of each village or in a school or in the nearby health facility within a village after a 2 days prior announcement and community mobilization. Two days prior to the mass blood investigation, mothers/caretakers were informed to bring her/his under-five children to the center. A unique card was given for each child during the announcement period and the child presented the card during the blood investigation. For malaria parasite identification, two thick and thin films were prepared from a finger prick in the field and stained with 3% Giemsa stain for 40-60 min in Jimma Specialized Hospital. Each slide was read by experienced laboratory technicians using 1,000 times oil immersion. Absence of malaria parasite in 200 high power ocular fields of the thick film was considered as negative [18]. For positive cases, type of species, degree of parasitaemia and presence of gametocytes were reported. Symptomatic malaria was defined as presence of fever (axillary temperature ≥ 37.5°C) and malaria parasite of any stages at the same time. Haemoglobin concentration was determined using Hemo Cue analyzer *(HemoCue Hb 301, Sweden*) in the field level. Weight and height of the under-five children were also measured during the mass blood investigations.

Focus Group Discussions (FGD) were also conducted to explore the perception of the community about the utilization of LLITN and burden of malaria. A total of eight FGD (four among men and four among women) were conducted in both the intervention and the control villages during the baseline survey. The same numbers of FGDs were conducted at the end of the project. The participants of the FGD were selected purposively in consultation with the *Kebele *leaders. All the FGD were tape recorded after consent was obtained from the participants.

### Data management and statistical analysis

Data were entered into computer, edited, cleaned, and analysed using SPSS-16 and STATA. Both unadjusted and adjusted effect sizes for the trial endpoints are presented. The adjusted results were presented after taking into account potential confounders such as age and sex. Crude and adjusted results were compared between the intervention and control villages at baseline, 6-months and 12-months of the project. Confidence intervals of percentages were calculated taking into account the villages as a cluster variable using weighted t-tests in STATA. Multivariate analysis was conducted using the simultaneous entry complex surveys logistic regression model by taking into account the clustering effect at the villages level. A P-value of less than or equal to 0.05 was considered to be significant for all tests. The presentation has also followed the CONSORT statement guidelines for cluster randomized trials [19].

Analysis of the qualitative data was done manually. All the Focus Group Discussions (FGD) notes and tap records were translated from Afan Oromo (local language) to English. The translated texts were read by the investigators several times and mutually exclusive and meaningful categories/themes were developed. The data were then coded according to the category system. Data belonging to each category were retrieved, assembled and viewed to get meaningful interpretation based on the objectives of the study. The interpreted qualitative data were presented together with the quantitative results to triangulate the findings. Individuals quotes were used to illustrate major findings.

### Ethical considerations

The proposal was approved by Jimma University and the WHO ethical committee. Written consent was obtained from caretakers of under-five children. Patients with anaemia and malaria were given standard [20] treatment by the health extension workers or nearby health centers.

## Results

### Socio-demographic characteristics

A total of 2410, 2037 and 2612 under-five children were included at the baseline, six and twelve months of the study respectively. At the baseline survey, the mean age of children were 33.5(SD ± 1.8) and 32.1(SD ± 1.75) months in the intervention and control arms, respectively. The intervention and control villages had no differences in terms of age and sex of the children. The age of the children in the intervention and control villages at the 6^th ^months of the survey was 34.22 (SD ± 21.14) and 32.95 (SD ± 15.55) months respectively (*p *= 0.086). Whereas at the 12th month, the mean age at the intervention and control villages was 34.31 (SD ± 15.66) and 34.82 (SD ± 21.16) months respectively (*p *= 0.535). The proportion of male and female children in the intervention and control villages was similar at baseline, 6^th ^and 12^th ^months of the project period (Table 1).

**Table 1:** Comparison of the intervention and control villages by age, sex, malaria parasite and anaemia, south-west Ethiopia*

Variable	Baseline		6 months		12 months	
	
	Control, n (%)	Intervention, n (%)	Control, n (%)	Intervention, n (%)	Control, n (%)	Intervention, n (%)
Age in months						
< 12	263(21.9)	246(20.4)	240 (17.6)	191 (15.3)	154 (16.1)	184 (17.1)
12-23	94(7.8)	62(5.1)	155 (11.4)	156 (12.5)	91 (9.5)	105 (9.7)
24-35	222(18.5)	244(20.2)	251 (18.4)	243 (19.4)	177 (18.5)	187 (17.3)
36-47	226 (18.8)	236(19.6)	249 (18.3)	227 (18.2)	188 (19.6)	216 (20.0)
> = 47	398 (33.1)	419 (34.7)	467 (34.3)	433 (34.6)	349 (36.4)	386 (35.8)
Total	1203	1207	1362	1250	959	1078
Sex						
Male	607 (50.5)	616 (51.0)	694 (51.0)	631 (50.5)	464 (48.4)	542 (50.3)
Female	596 (49.5)	591 (49.0)	668 (49.0)	619 (49.5)	495 (51.6)	536 (49.7)
Total	1203	1207	1362	1250	959	1078
Malaria parasite						
Positive	100 (8.3)	127 (10.5)	176 (12.9)	81 (6.5)	64 (6.7)	67 (6.2)
Negative	1103(91.7)	1080 (89.5)	1186 (87.1)	1169 (93.5)	893 (93.3)	1011 (93.8)
Total	1203	1207	1362	1250	957	1078
Malaria parasite species^§^						
*P.falciparum*	38 (38.0)	52 (40.9)	135 (76.7)	43 (53.1)	45 (66.2)	33 (50.0)
*P. vivax*	60 (60.0)	75 (59.1)	41 (23.3)	38 (46.9)	23 (33.8)	33 (50.0)
Mixed infection	2 (2.0)	0 (0.0)	0 (0.0)	0 (0.0)	0 (0.0)	0 (0.0)
Total	100	127	176	81	64	66
Symptomatic malaria						
Yes	36 (3.0)	52 (4.3)	158 (11.6)	57 (4.6)	28 (2.9)	17 (1.6)
No	1167 (97.0)	1155 (95.7)	1204 (88.4)	1192 (95.4)	929 (97.1)	1061 (98.4)
Parasite Density						
+1	37 (82.2)	71 (86.6)	82 (60.3)	29 (50.9)	27 (58.7)	25 (59.5)
+2	4 (8.9)	8 (9.8)	38 (27.9)	24 (42.1)	14 (30.4)	17 (40.5)
+3	4 (8.9)	3 (3.6)	16 (11.7)	4 (7.0)	6 (10.8)	0 (0.0)
Total	45	82	136	57	47	42
Presence of fever						
Yes	304(25.3)	380(31.5)	1060 (77.8)	670 (53.6)	328 (34.2)	316 (29.7)
No	899(74.7)	827(68.5)	302 (22.2)	579 (46.4)	631 (65.8)	762 (70.3)
Total	1203	1207	1362	1249	959	1078
Anaemia						
Yes (< 11 g/dl)	428(35.6)	352(29.2)	727 (54.1)	616 (51.2)	446 (46.8)	441 (41.6)
No	775(64.4)	855(70.8)	616 (45.9)	587 (48.8)	507 (53.2)	620 (58.4)
Total	1203	1207	1343	1203	953	1061
Severe anaemia						
Yes (Hgb < 8 g/dl)	52 (4.4)	52 (3.3)	85 (6.3)	84 (6.9)	49 (5.1)	46 (4.3)
No	1140 (95.6)	1515 (96.7)	1268 (93.7)	1136 (93.1)	915 (94.9)	1026 (95.7)
Total	1192	1567	1353	1220	964	1072

*Figures in cells are raw percentages, without weighting for cluster sizes; and percentages are calculated based on non-missing responses for the variables concerned

**^§ ^**The denominator is total positive malaria cases, Hgb = Haemoglobin

### Burden of malaria and anaemia at the 6 months of the intervention

**Figure 2:**
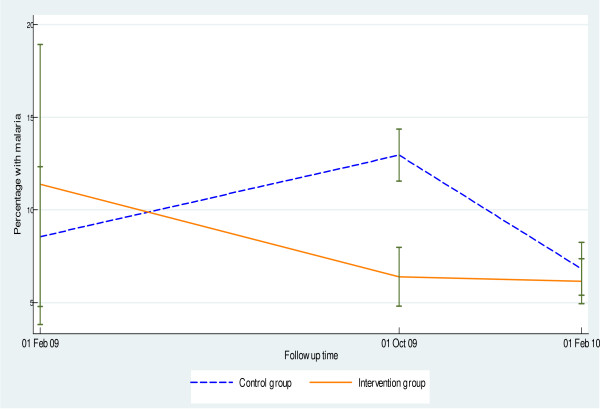
**Percentage of under-five children with malaria at the different periods of the project**.

The prevalence of malaria has decreased by 38% (from 10.5% to 6.5%) at the 6th months of the project period in the intervention villages. On the other hand, the prevalence has steadily increased by 55% in the control villages during the same period (Figure 2). Similarly, symptomatic malaria has steadily declined in the intervention villages compared to the control villages (Table 1).

During the high transmission/epidemic season at the 6^th ^months of the project period, children in the intervention arm were less likely to have malaria as compared to the children in the control arm (OR = 0.42; 95%CI: 0.32,0.57). On the other hand, children in the intervention arm were less likely to have symptomatic malaria compared to children in the control arm (OR = 0.33; 95%CI: 0.24, 0.46) (Table 2).

**Table 2:** Comparison of burden of malaria and anaemia between the intervention and control villages, south-west Ethiopia

Variables	**Control (N = 11)**, % (SD)	**Intervention (N = 11)**, % (SD)	Mean/proportion Difference % (95% CI)	*p*-value	Intervention vs. Control Adjusted OR (95% CI)*
Malaria					
Baseline	8.6(3.1)	10.5 (5.6)			
6 months	12.9(1.93)	6.5 (1.4)	-6.4(-8.7, -4.4)	0.000	0.42 (0.32-0.57), *p *value = 0.000
12 months	6.7 (1.5)	6.2 (0.94)	-0.5 (-2.5, 1.2)	0.426	0.98 (0.67-1.42), *p *value = 0.914
Presence of fever					
Baseline	25.3 (7.5)	31.5 (4.6)			
6 months	77.8 (10.17)	53.6 (12.8)	-24.2(-43.9,-4.5)	0.023	0.35 (0.29-0.42) *p *value = 0.000
12 months	34.2 (11.0)	29.3 (10.0)	-4.9 (-22.4, 12.6)	0.530	0.79 (0.65-0.97), *p *value = 0.023
Symptomatic malaria					
Baseline	3.0 (1.93)	4.3 (2.9)			
6 months	11.6 (1.6)	4.6(1.0)	-7.0 (-8.6,-5.5)	0.000	0.33 (0.24-0.46), *p *value = 0.000
12 months	2.9(0.9)	1.6 (0.6)	-1.3 (-2.6,-0.1)	0.039	0.49 (0.26*-*0.94), *p *value = 0.033
Moderate anaemia (Hgb < 11 g/dl)					
Baseline	35.6 (5.5)	29.2 (2.9)			
6 months	54.1 (6.6)	51.2 (8.9)	-2.9 (-15.3, 9.2)	0.579	0.84 (0.71-0.99), *p *value = 0.043
12 months	46.8 (3.1)	41.6 (8.7)	-5.2 (-17.5, 6.9)	0.335	0.73 (0.60-0.89), *p *value = 0.002
Severe anaemia (Hgb < 8 g/dl)					
Baseline	4.4 (1.70)	3.32 (1.33)			
6 months	6.3 (1.04)	6.88 (1.62)	0.60 (-1.41, 2.62)	0.502	1.13 (0.81, 1.58), *p *value = 0.472
12 months	5.0 (2.07)	3.96 (2.36)	-1.07 (-4.46, 2.31)	0.478	0.91 (0.73, 1.14), *p *value 0.432

*Adjusted for age and sex and clustering in the logistic regression model, Hgb = Hemoglobin

The prevalence of moderate anaemia became higher at the sixth months of the follow up period in both groups. Nonetheless, children in the intervention arm were less likely to be anemic than those in the control arm (OR = 0.84; 95% CI: 0.71, 0.99). The prevalence of severe anaemia also showed an increment in the intervention and control groups though there was no statistically significant difference between the groups (OR = 1.13; 95% CI: 0.81 - 1.58).

### Burden of malaria and anaemia at 12th month of the intervention

The prevalence of malaria declined in both villages at the 12th month (Figure 2). Although not statistically significant, malaria prevalence was higher at the control villages compared to the intervention ones (proportion difference = -0.5; 95%CI: -2.5, 1.2). There was decline in the prevalence of symptomatic malaria in both the control and intervention villages. Children in intervention arm were less likely to have symptomatic malaria than children in the control arm (OR = 0.49; 95%CI: 0.26, 0.94).

The prevalence of moderate anaemia in under-five children was lower in the intervention villages than the control villages (OR = 0.73; 95%CI: 0.60, 0.89). However, the two groups were not different in terms of severe anaemia (Table 2).

The qualitative findings were in line with the quantitative measurements. The community in the intervention villages acquired significant skill and knowledge to utilize LLITN properly and consistently. A 50 years-old farmer in the intervention villages discussed the proper utilization of LLITN as:

"*They gave us enough bed nets and repeatedly taught us how to hang and put it under the bed. All village members are now aware of using bed nets."*

On the other hand, community members in the control villages complained about the lack of skills and knowledge how to use the LLITN in their *Tukul *houses. For instance, a 25 years-old discussant said, *"Although we received enough bed nets, people did not know how to hang and use it properly. Because of lack of knowledge, most people use the bed nets improperly such as sleeping on it."*

The qualitative finding revealed that the burden of malaria varied between the intervention and control villages. Most FGD participants in the intervention villages believed that the proper and consistent use of LLITN had significantly reduced the burden of malaria in their villages. For instance, a 55 years old lady said, *"Previously malaria was very common and it highly affected our family and neighbours, however, the distribution of bed nets and the constant training had saved our lives."*

In the control villages, people perceived that the burden of malaria was still very high in most of the villages. However, the female discussant in some villages expressed that malaria has declined due to the distribution of bed nets and the occasional health education efforts of the health extension workers.

## Discussion

This cluster randomized trial reveals that training of the household heads on the use of LLITN has great impact on the reduction of malaria burden particularly at high transmission seasons. In Ethiopia, the peak season of malaria incidence is from September to December every year. This study showed that children in the intervention villages had a 0.42 times higher odds of developing malaria compared to those who reside in the control villages. Symptomatic malaria was also highly reduced in the intervention arms compared to the control villages. In a country like Ethiopia where malaria is the first cause of death among under-five children, scale up of such an intervention would have far reaching impact on malaria incidence and to achieve the malaria related Millennium Development Goal.

Previous studies have also documented the impact of LLITN on reduction of the burden of malaria. In western Kenya where there is an intense transmission, 19% reduction in *P. falciparum *prevalence was observed due to LLITN [21]. Other studies have also documented the impact of insecticide-treated bed nets on the prevalence of malaria [22,23]. The current study also indicated that children in the intervention villages were less like to be febrile during high transmission seasons. Previous studies conducted in western Kenya reported a reduction of 38% in febrile episodes in all age groups [23]. Other non-randomized controlled studies in pre-school children in western Kenya and Tanzania during an intense malaria transmission also found a 60% reduction in febrile episodes as a result of LLITN utilization [24-26].

There has been considerable debate about how to sustain use of LLITN for better impact [27,28]. Involvement of the community at the grass root level and empowering them is one means to sustain LLITN utilization. Sustainable utilization of LLITN is particularly important in places like Gilgel Gibe where the prevalence of malaria is very high due to possible ecological disruption. The prevalence of malaria among children less than five years of age at the different phases of this study was higher than previous reports in Ethiopia [29]. A multi-regional study by Shargie et al. in Ethiopia showed a lower (4.6%) prevalence rate of malaria in under-five children [29]. Recent survey also showed a much lower prevalence of malaria (0.6%) in other parts of Ethiopia [30]. According to the Ethiopian malaria indicator survey of 2007, the prevalence of Malaria in under-five children was 0.9% [31]. Malaria in southwest Ethiopia around the Gilgel Gibe field research center has been constantly high since 2005 due to the possible ecological disruption [17].

Malaria transmission in Ethiopia is temporally and spatially dynamic, with unstable and seasonal transmission linked to environmental variables such as altitude and rainfall [32]. The burden of malaria peaks twice a year, after the two rainy seasons (March-May and July-October) [33]. However, reports indicate that in Oromiya regional state where GGFRC is located; there is one annual peak of transmission from September to December [34]. According to the Federal Ministry of Health and the World Health Organization (WHO) reports, classification of the study area is under highland fringe with low unstable transmission [30].

The prevalence of anaemia and malaria was high during the high transmission season. However, children in the intervention villages were 16% less likely to be moderately anaemic which is consistent with previous reports [9,18,35]. The causes of anaemia in Africa are complex and multi-faceted. Previous report in the same area [36] indicated that there was a strong association between malaria and anaemia. This indicates that prevention of malaria through utilization of LLITN could reduce the burden of anaemia among vulnerable groups.

Unlike other cluster randomized trials, the current intervention focuses on community empowerment through training on the utilization LLITN to reduce the burden of malaria. In the absence of such capacity building and self-reliant strategies, distribution of LLITN will not be effective and efficient. This study, however, did not assess the sustainability of such community empowerment strategy after the end of the project and its cost effectiveness in the long term. This cluster randomized trial was done in a community at higher risk of malaria and as such may not be generalizable to other settings.

In conclusion, this study indicates that training of household heads in the community could increase the uptake of LLITN and significantly reduces the burden of malaria and anaemia in areas with unstable malaria transmission during peak transmission season. The ministry of health (MOH) in collaboration with other partners should design similar strategies to reduce the burden of malaria in high risk communities.

## Competing interests

The authors declare that they have no competing interests.

## Authors' contributions

AD conceived the study and was involved in the design, coordination, field supervision, analysis and drafted the manuscript. AR, TD and FT were involved in the data analysis and reviewed the article. LS and FA participated in the design, field supervision and report writing. ZB and MS were involved in field supervision and writing of the qualitative report. AZ was involved in the laboratory quality control and field supervision. KD and SB drafted and reviewed the article. All authors read and approved the manuscript.
